# CD80 as an Important Immune Mediator and Therapeutic Target in Atherosclerosis

**DOI:** 10.3390/ijms27188215

**Published:** 2026-09-15

**Authors:** Alexander Blagov, Aleksey Vatlin, Daria Borodko, Anastasia Maksaeva

**Affiliations:** Laboratory of Molecular Genetic Modeling of Inflammaging, Institute of General Pathology and Pathophysiology, 8, Baltiiskaya Street, 125315 Moscow, Russia

**Keywords:** CD80, atherosclerosis, T cells, CD86

## Abstract

Atherosclerosis is a chronic immune–inflammatory disease of the arterial wall in which antigen-presenting cells (dendritic cells, macrophages and B cells) present locally generated antigens, such as oxidized low-density lipoprotein, to plaque-infiltrating T cells. Full T-cell activation requires costimulation through the B7 family ligands CD80 (B7-1) and CD86 (B7-2), which engage CD28 to activate T cells and cytotoxic T-lymphocyte-associated protein 4 (CTLA-4) to restrain them. Although long considered functionally redundant, CD80 and CD86 are now known to be structurally and mechanistically distinct, differing in receptor affinity, susceptibility to CTLA-4-mediated trans-endocytosis, capacity to cis-heterodimerize with programmed death-ligand 1, and dependence relationships with regulatory versus effector T cells. In atherosclerosis, CD80 is expressed by lesional dendritic cells, macrophages, foam cells, B cells and, under inflammatory conditions, vascular endothelial and smooth muscle cells, with expression levels correlating with plaque vulnerability in humans. Genetic deletion, CTLA-4-Ig-based pharmacological blockade and selective small-molecule antagonism in preclinical models generally converge on a proatherogenic role for CD80/CD86-dependent costimulation, although combined genetic deletion of CD80 and CD86 has also been reported to paradoxically enhance early lesion formation in some contexts, while augmented CTLA-4-mediated coinhibition is atheroprotective—a balance corroborated by the accelerated atherosclerosis and cardiovascular toxicity observed with checkpoint-inhibitor cancer immunotherapy. Molecular imaging agents targeting CD80/CD86, repurposed CTLA-4-Ig biologics such as abatacept, and antigen-specific tolerogenic dendritic cell strategies together constitute an emerging translational pipeline. To our knowledge, this is the first review to integrate CD80’s structural and receptor pharmacology with its cellular sources in the plaque, preclinical genetic and pharmacological evidence, human plaque and biomarker data, molecular imaging approaches, and checkpoint inhibitor-associated cardiovascular toxicity within a single translational framework. This review synthesizes the structural biology, cellular sources, preclinical genetics, human plaque and biomarker data, and therapeutic and diagnostic implications of CD80 as an immune mediator and candidate therapeutic target in atherosclerosis.

## 1. Introduction

Atherosclerosis remains the principal pathological substrate of ischemic heart disease, ischemic stroke and peripheral arterial disease, and its global burden continues to expand as populations age. Global Burden of Disease estimates place the worldwide cardiovascular disability-adjusted life-year (DALY) total at 437 million in 2023, a 1.4-fold rise since 1990, with cardiovascular deaths increasing from 13.1 million to 19.2 million over the same interval [1]; projections through 2050 anticipate a further 90% rise in cardiovascular prevalence [2], driven substantially by atherosclerotic disease [3,4,5]. Once viewed primarily as a disorder of lipid deposition, atherosclerosis is now firmly established as a chronic immune–inflammatory disease of the arterial wall, in which both innate and adaptive immunity orchestrate lesion initiation, progression and, ultimately, plaque instability [6,7,8,9,10]. Antigen-presenting cells (APCs)—dendritic cells (DCs), macrophages and, to a lesser extent, B cells—infiltrate the subendothelial space together with monocytes and lymphocytes, where they process locally generated neo-antigens such as oxidized low-density lipoprotein (oxLDL) and heat-shock proteins and present them to T cells [11,12]. Full T-cell activation, however, requires more than antigen recognition through the T-cell receptor (TCR)–major histocompatibility complex (MHC) interaction: a second, costimulatory signal delivered by the B7 family of ligands on APCs is obligatory for productive T-cell responses [13].

CD80 (B7-1) was first described as a B-cell activation antigen before its costimulatory role in T-cell proliferation and IL-2 production was defined [14,15]. Together with its close paralogue CD86 (B7-2), cloned as a second CD28/CTLA-4 counter–receptor [16], CD80 engages the receptors CD28 and cytotoxic T-lymphocyte-associated protein 4 (CTLA-4, CD152) on T cells to deliver opposing—activating and inhibitory—signals [17,18,19]. Landmark functional work established that CD28 and CTLA-4 have opposing effects on T-cell responses despite sharing ligands, identifying CTLA-4 as a dominant negative regulator rather than a further costimulator [20,21]. Although CD80 and CD86 were long regarded as functionally redundant, an accumulating body of structural, biochemical and immunological evidence indicates that they are mechanistically distinct: they differ in affinity for CD28 and CTLA-4, in their capacity to heterodimerize with programmed death-ligand 1 (PD-L1), in their susceptibility to CTLA-4-mediated trans-endocytosis, and in the cellular contexts in which they are expressed [22,23,24]. These distinctions have direct relevance to atherosclerosis, where CD80 and CD86 are co-expressed on lesional macrophages and DCs but appear to make quantitatively and qualitatively different contributions to plaque T-cell priming, plaque vulnerability, and the balance between effector and regulatory T-cell responses [25,26].

This review synthesizes current knowledge of CD80 biology in atherosclerosis: its molecular structure and receptor interactions; cellular sources within the lesion; genetic and pharmacological manipulation in preclinical models; behavior in human plaque tissue and blood; and therapeutic and diagnostic strategies—from CTLA-4-Ig fusion proteins and small-molecule B7-1 antagonists to CD80-targeted molecular imaging. Because CD80 sits at a nodal point between costimulation, coinhibition and cis-interactions with PD-L1, understanding its atherosclerosis-specific biology matters both for interpreting cancer-immunotherapy cardiovascular toxicity and for designing vascular-selective immunomodulatory therapies [27,28].

This narrative review is based on a structured literature search of the PubMed/MEDLINE, Scopus and Web of Science databases, performed through August 2026, using combinations of the terms “CD80”, “B7-1”, “CD86”, “CTLA-4”, “PD-L1”, “atherosclerosis”, “costimulation” and “immune checkpoint”, supplemented by manual screening of the reference lists of retrieved articles; no lower publication-date limit was applied, given the continued relevance of foundational immunological studies from the 1980s and 1990s cited throughout this review.

## 2. Molecular Biology of CD80: Structure, Ligands and Signaling

CD80 is a type I transmembrane glycoprotein of the immunoglobulin superfamily, comprising an extracellular region with one membrane-distal V-set and one membrane-proximal C2-set Ig-like domain, a single transmembrane segment, and a short cytoplasmic tail; it shared a fold with CD86 and was resolved crystallographically for both receptor-binding domains [29,30,31]. It was originally identified as the ligand providing “signal two” for T-cell activation, and CD80 and CD86 were subsequently shown to provide broadly similar costimulatory signals for T-cell proliferation, cytokine production and cytotoxic T-lymphocyte generation when expressed on transfected cell lines [18]. Despite this apparent functional overlap, CD80 and CD86 share only approximately 25–30% amino-acid sequence identity, arise from an ancient gene-duplication event, and have since diverged substantially in their biophysical and cell-biological behavior [22]. Early tissue-distribution studies in the rat demonstrated that, in contrast to CD86, which is broadly expressed by non-lymphoid interdigitating and splenic dendritic cells as well as germinal-center B cells, CD80 is largely undetectable in resting lymphoid tissue and is instead induced upon activation, an expression pattern subsequently confirmed in mouse and human systems and consistent with CD80 functioning as a marker of APC activation rather than a constitutive costimulatory ligand [32].

### 2.1. CD28 and CTLA-4: Opposing Receptors, Shared Ligands

Naïve T-cell activation requires simultaneous engagement of the TCR–peptide-MHC complex and CD28 by CD80 or CD86; in the absence of this second signal, T cells become anergic or die rather than proliferate [13,17]. CTLA-4, which is upregulated on activated T cells and constitutively expressed by regulatory T cells (Tregs), binds the same ligands with substantially higher avidity than CD28 and delivers an inhibitory signal, both by directly antagonizing CD28 signaling and by physically removing CD80/CD86 from the APC surface through trans-endocytosis [33,34]. CD80 forms a high-avidity, bivalent “dimer-of-dimers” lattice with CTLA-4 that results in stable binding, extensive CTLA-4 ubiquitylation and lysosomal degradation of the CTLA-4–CD80 complex, whereas CD86 forms a lower-affinity, pH-sensitive interaction that permits CTLA-4 recycling back to the cell surface for further rounds of ligand capture [22]. This asymmetry means that CD80 is intrinsically the more potent driver of CTLA-4-mediated ligand depletion, while CD86 is comparatively more important for sustaining sequential rounds of CTLA-4-dependent immune regulation—an asymmetry with direct implications for how CD80 blockade versus CD86 blockade might be expected to affect vascular inflammation.

### 2.2. CD80–CD86 Functional Divergence on T Cells and Tregs

CD80 and CD86 also differ in the T-cell subsets that depend upon them. In the presence of high constitutive CTLA-4 expression, CD80–CD28 interactions on Tregs are selectively impaired, such that CD86 becomes the dominant ligand supporting the Treg proliferation, survival and expression of CTLA-4, ICOS and OX40 [35]. Conversely, activated conventional CD4+ T cells can themselves express CD80 or CD86 in a CD28-costimulation-dependent manner: CD80+ T cells acquire a FoxP3hi, CTLA-4hi induced-Treg-like phenotype with limited proliferation, whereas CD86+ T cells adopt an interferon-γ-producing effector phenotype with greater proliferative capacity [36]. CD80 expressed by Tregs can further act as an intrinsic CTLA-4 ligand, reinforcing Treg homeostasis through autocrine or juxtacrine CD28 signaling [36]. Because atherosclerosis is characterized by an imbalance favoring proatherogenic effector and Th1/Th17 responses over atheroprotective Treg responses, this CD80/CD86 dichotomy in T-cell and Treg biology provides a mechanistic rationale for the divergent atherogenic phenotypes reported when CD80 alone, CD86 alone, or both are genetically deleted in mouse models (Section 4). Live-cell imaging shows CD28–CD80 interactions specifically control Treg motility and synapse stability: Tregs form stable DC conjugates before, but not after, downmodulating DC CD80 [37]. Selective CD28 antagonism (versus CTLA-4 or PD-L1 blockade) increases Treg dwell time and suppressive function while dampening effector responses, showing the CD80/CD86–CD28/CTLA-4 axis can be pharmacologically dissected toward regulatory outcomes [38]. Treg-specific depletion models confirm Tregs are the principal controllers of steady-state CD80/CD86 density on APCs in vivo [39], and in Chagas cardiomyopathy, distinct CD80- versus CD86-dependent CD28/CTLA-4 interactions differentially modulate Treg activity, reinforcing this dichotomy’s relevance beyond atherosclerosis [40].

### 2.3. CD80–PD-L1 Cis-Interactions: A Third Layer of Regulation

**Figure 1:**
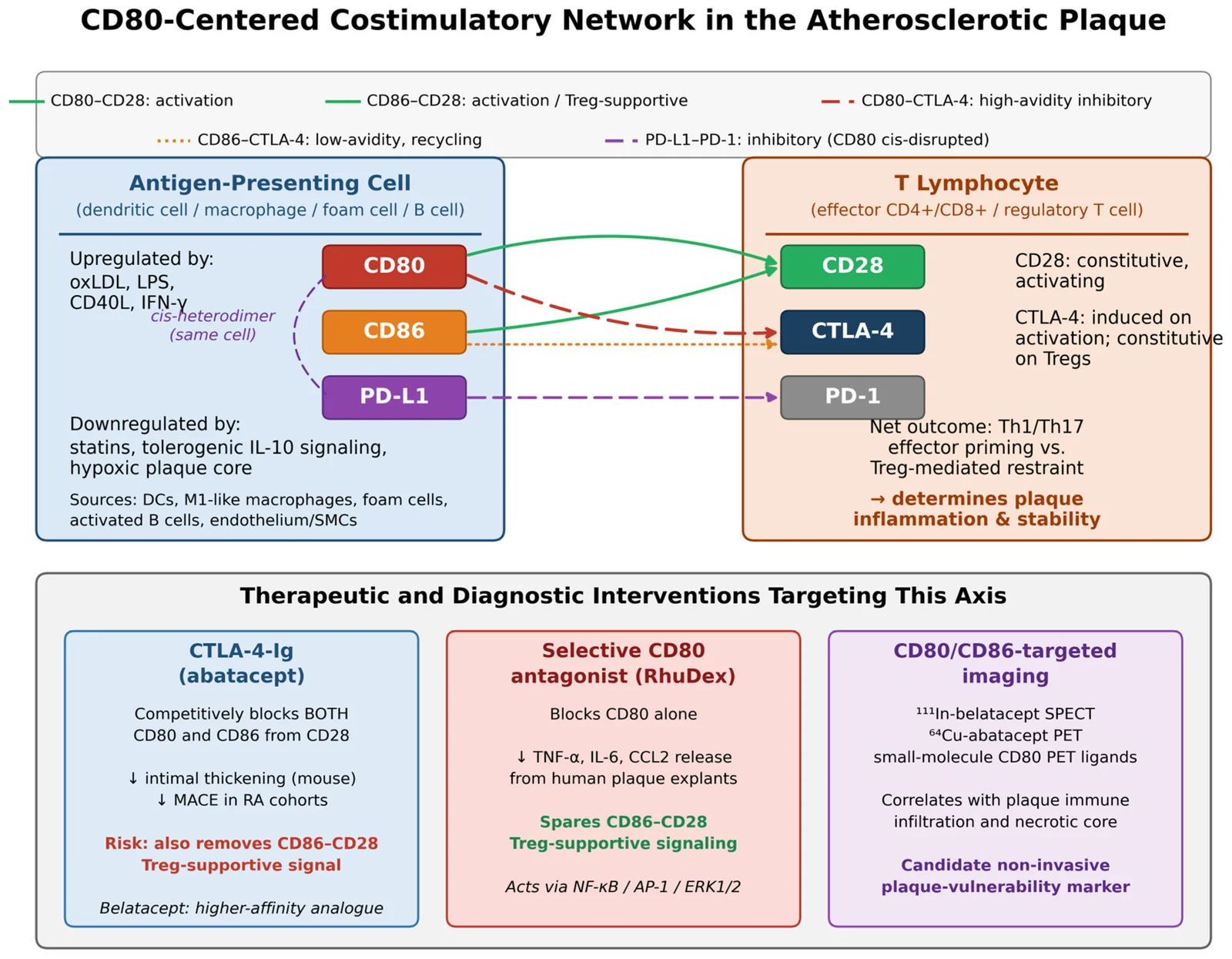
CD80-centered costimulatory network in the atherosclerotic plaque: receptor–ligand interactions (CD80/CD86–CD28/CTLA-4, PD-L1–PD-1 and the PD-L1:CD80 cis-heterodimer) and current therapeutic/diagnostic strategies targeting this axis.

A further, more recently appreciated layer of CD80 biology involves its cis-heterodimerization, on the surface of the same APC, with PD-L1. This interaction was originally reported to occur in trans between opposing cells, but more rigorous biophysical and cell-biological analyses demonstrated that PD-L1 and CD80 preferentially heterodimerize in cis on APCs, and that this heterodimer disrupts both PD-L1–PD-1 and CD80–CTLA-4 trans-interactions while leaving CD80–CD28 costimulation intact [23,24,41]. Mechanistically, PD-L1:CD80 cis-heterodimerization reduces CD80 homodimerization, which in turn disrupts CD80–CTLA-4 engagement and subsequent CTLA-4-mediated trans-endocytosis of CD80, and additionally sequesters PD-L1 away from PD-1 [41,42]. Knock-in mice bearing mutations that selectively disrupt the PD-L1–CD80 cis-interface, without affecting CD80–CD28/CTLA-4 binding, show impaired anti-tumor and autoimmune T-cell responses, formally establishing the cis-interaction as the physiologically dominant mode of PD-L1–CD80 engagement on activated APCs in inflamed tissue [43]. The CTLA-4 immune checkpoint protein has itself been shown to regulate PD-L1–PD-1 interactions indirectly, via selective trans-endocytosis of CD80 that liberates PD-L1 for PD-1 engagement, again demonstrating specific removal of CD80 while its PD-L1 heterodimeric partner remains on the APC surface [44]. In sum, CD80 functions as a molecular hub that integrates at least three checkpoint axes—CD28 costimulation, CTLA-4 coinhibition and PD-L1/PD-1 signaling—and the relative abundance of CD80, CD86 and PD-L1 on a given APC determines the net output of antigen presentation (Figure 1). This biochemical complexity is directly relevant to atherosclerotic plaques, in which myeloid cells co-express CD80, CD86 and PD-L1 at substantial, and sometimes discordant, levels [25].

## 3. Cellular Sources of CD80 in the Atherosclerotic Vessel Wall

### 3.1. Dendritic Cells

Vascular DCs are present even in the healthy arterial intima and accumulate progressively as atherosclerosis develops, forming clusters with T cells that are consistent with local, intraplaque antigen presentation rather than antigen presentation confined to draining lymph nodes [11,12]. Detailed phenotypic mapping of aortic DC subsets shows that plasmacytoid DC depletion accelerates plaque growth via increased T-cell proliferation and interferon-γ, whereas dietary eicosapentaenoic acid expands a tolerogenic, CD80-low/CD86-low splenic DC population—linking CD80/CD86-low DC phenotypes to atheroprotection [45]. Mature DCs, identified by CD83 expression, characteristically co-express CD80 and CD86, and quantitative PCR and immunohistochemical analyses of human carotid endarterectomy specimens have shown that CD11c, CD80, CD83 and CD86 transcripts are significantly higher in unstable, symptomatic plaques than in stable lesions, with the increase being most pronounced in the mature-DC subpopulation [12,46]. Distinct DC subsets appear to exert opposing effects: CD11b+ conventional DCs, plasmacytoid DCs (pDCs) and CCL17+ DCs have each been linked to proatherogenic activity, and CCL17+ DCs in particular display elevated CD40, CD80 and CD86, consistent with in situ activation of CD4+ T cells within the plaque, whereas CD103+ DCs appear to be atheroprotective [7]. Because CD80 and CD86 are also expressed by many tissue macrophage subsets, unambiguous discrimination between DCs and macrophages in the non-lymphoid arterial wall on the basis of these markers alone remains difficult, and this ambiguity has practical consequences for interpreting immunohistochemical and flow-cytometric CD80 data from human and murine lesions [11].

### 3.2. Macrophages and Foam Cells

CD80 is classically regarded as a marker of classically activated, proinflammatory M1-polarized macrophages in the mouse, in parallel with CD86 as the corresponding human M1 marker, although this dichotomy is imperfect: human polarized macrophages respond differently to oxLDL depending on prior M1/M2 polarization state [47], and human macrophages exposed to oxLDL or native LDL can adopt hybrid, “Mox”-like phenotypes that co-express pro- and anti-inflammatory features [48,49]. Early immunohistochemical work established that both CD80 and CD86 are expressed on lesional macrophages in human arterial plaques and colocalize with oxLDL deposits, a putative autoantigen for T-cell activation within the lesion [50,51]. In murine ApoE-deficient and low-density lipoprotein receptor-deficient (*Ldlr*−/−) atherosclerosis models, CD80 and CD86 immunostaining is present from early through advanced stages of lesion maturation, again colocalizing with oxLDL [50]. At the single-cell level, unpolarized human macrophages express low surface CD80 associated with high CD206 (mannose receptor), while classical M1 polarization with lipopolysaccharide markedly upregulates CD80, and hypoxic or pathologic microenvironments—both features of the necrotic, poorly vascularized plaque core—reduce CD80 and CD86 expression together with antigen-uptake capacity, potentially limiting local T-cell triggering in the most advanced lesions [52]. This is consistent with large human carotid cohorts showing that other activation-associated macrophage markers, such as CD163, are similarly enriched in symptomatic, vulnerable plaques, indicating that costimulatory-ligand-high myeloid activation co-occurs with other markers of lesion instability [53].

### 3.3. B Cells and Germinal-Center Responses

B cells contribute to atherosclerosis both protectively, through B1-cell-derived natural IgM, and pathogenically, through follicular B2-cell differentiation into antibody-secreting plasma cells within germinal centers [54,55]. CD86 is preferentially expressed on memory and germinal-center B cells, whereas resting B cells express little CD80, but activation—including CD40 engagement by CD40L on cognate follicular helper T cells—upregulates both CD80 and CD86 and licenses full CD28-dependent T-cell help [54,56]. Pharmacological CD80/CD86 blockade with the CTLA-4-Ig fusion protein abatacept reduces plasmablast output and antigen-specific memory B-cell formation in humans, indicating that the germinal-center consequences of costimulatory blockade—relevant to circulating oxLDL-specific and other atherosclerosis-associated antibody titers—extend beyond direct T-cell effects [57].

### 3.4. CD8+ T Cells and Cross-Presentation

CD8+ T cells contribute substantially to macrophage cytotoxicity, necrotic-core expansion and vascular smooth muscle cell dedifferentiation, and their activation, like that of CD4+ T cells, is subject to CD80/CD86-dependent checkpoint control [58,59]. A comprehensive review of T-cell subsets notes that CD80/CD86–CD28 signaling drives proatherogenic Th1 responses (suppressed by CD80/CD86 deficiency), while the same ligands engage CTLA-4 on Tregs, placing CD80/CD86 at the convergence of antagonistic T-cell programs [60]. Notably, Batf3-dependent ablation of cross-presenting CD8α+ DCs sharply reduces CD8+ T-cell cross-priming without altering lesion burden or stability, suggesting other CD80/CD86-competent APCs suffice to sustain plaque T-cell activation [61].

### 3.5. Endothelial and Vascular Smooth Muscle Cells

Non-hematopoietic vascular cells can also upregulate CD80/CD86 and CD40 under inflammatory conditions, extending local T-cell costimulation beyond professional APCs to the endothelium and activated smooth muscle cells [29,62]. Statins reduce CD40/CD40L expression on endothelial cells, smooth muscle cells and macrophages in a concentration-dependent, mevalonate-reversible manner—a pathway upstream of, and permissive for, CD80/CD86 induction, since CD40 engagement is a well-established trigger for CD80/CD86 upregulation [62,63].

## 4. Genetic and Pharmacological Evidence from Preclinical Models

Costimulatory and coinhibitory pathway manipulation has been studied extensively across *Ldlr*−/− and ApoE−/− atherosclerosis models, with reviews cataloging OX40-OX40L, CD137-CD137L, CD28-CD80/CD86 and CD40-CD40L as generally proatherogenic and CTLA4-CD80/CD86, CD27-CD70 and PD-1-PD-L1/2 as generally atheroprotective [64,65].

Receptors and ligands that form the stimulatory or inhibitory molecular axis of action on CD80 are summarized in Table 1.

A methodological caveat applies throughout this section: most of the genetic and pharmacological evidence reviewed below derives from manipulations that remove or block CD80 and CD86 together (combined knockout mice, CD28 blockade, or CTLA-4-Ig, which binds both ligands) rather than from CD80-selective interventions. Where a finding reflects this joint CD80/CD86 manipulation, we describe it as such; the more limited evidence bearing specifically on CD80 in isolation—chiefly the single-cell atlas data (Section 5.1) and the RhuDex explant studies (Section 4.4 and Section 8.2)—is flagged explicitly.

### 4.1. Loss-of-Function Studies

The clearest genetic demonstration that CD80/CD86 costimulation is proatherogenic comes from Buono and colleagues, who showed that combined deletion of CD80 and CD86 in *Ldlr*−/− mice paradoxically enhanced, rather than reduced, early lesion formation at an early (8-week) timepoint on a 1.25% cholesterol diet, assessed in the aortic root and arch, an effect not sustained at a later (20-week) timepoint while altering plaque antigen-specific T-cell responses to the atherosclerosis-associated antigen heat-shock protein 60 (HSP60), establishing B7-1/B7-2 costimulation as a direct regulator of plaque antigen-specific T-cell priming and atherogenesis [26,66]. Subsequent work using a femoral artery cuff model of post-interventional vascular remodeling found that both CD4−/− mice and CD80−/−CD86−/− mice developed significantly smaller intimal lesions than wild-type controls, directly implicating the CD28–CD80/CD86 axis in CD4+ T-cell-dependent vascular remodeling after mechanical injury [67]. In graft-arterial-disease models—mechanistically related to accelerated, immune-driven arteriopathy—mice lacking B7-1, B7-2, or both similarly show altered graft vasculopathy, reinforcing a general role for CD80/CD86 in immune-mediated arterial remodeling beyond native atherosclerosis [68].

**Table 1:** Costimulatory and coinhibitory ligand–receptor pairs relevant to CD80 biology in atherosclerosis.

Ligand	Receptor	Signal Type	Net Effect on Atherogenesis	Key Refs
CD80 (B7-1)	CD28	Activating (costimulatory)	Proatherogenic—drives effector T-cell priming	[26,67]
CD80 (B7-1)	CTLA-4 (CD152)	Inhibitory—high avidity; drives trans-endocytosis	Atheroprotective when engaged	[22,33]
CD86 (B7-2)	CD28	Activating; Treg-supportive	Context-dependent; sustains Treg homeostasis	[35]
CD86 (B7-2)	CTLA-4 (CD152)	Inhibitory—lower avidity; ligand recycling	Atheroprotective	[22,35]
PD-L1	PD-1 (trans)	Inhibitory	Atheroprotective; loss accelerates atherosclerosis	[23,24]
PD-L1	CD80 (cis, same cell)	Disrupts PD-L1–PD-1 and CD80–CTLA-4 trans binding	Modulatory; net effect context-dependent	[23,24,43]
CD40L (CD154)	CD40	Upstream inducer of CD80/CD86 expression	Proatherogenic	[62,63]
OX40L	OX40	Activating	Proatherogenic	[64,65]
CD137L	CD137 (4-1BB)	Activating	Proatherogenic	[64,65]
CD70	CD27	Context-dependent	Generally atheroprotective (Treg-associated)	[64,65]

Abbreviations: CTLA-4, cytotoxic T-lymphocyte-associated protein 4; PD-1, programmed cell death protein 1; PD-L1, programmed death-ligand 1. Net-effect entries for CD80 (B7-1) reflect studies in which CD80 and CD86 were manipulated jointly (e.g., combined knockout, CD28 or CTLA-4-Ig blockade) unless a CD80-selective intervention is specified.

### 4.2. CTLA-4-Ig and CTLA-4 Overexpression: Pharmacological and Genetic Coinhibition

If CD80/CD86-CD28 costimulation promotes atherogenesis, augmenting coinhibition should be protective, and this has been confirmed by multiple approaches. Systemic abatacept—a soluble CTLA-4-Ig fusion protein competitively blocking CD28–CD80/CD86 engagement—reduced post-interventional intimal thickening by 58.5% in the femoral cuff model and accelerated atherosclerosis by 78.1% in hypercholesterolemic ApoE3*Leiden mice, with reduced splenic CD4+ T-cell activation markers, lower interferon-γ and higher interleukin-10 [67]. In a homocysteine-accelerated atherosclerosis model, CTLA4-Ig similarly ameliorated disease by inhibiting T-cell overactivation in *ApoE*−/− mice [69]. Complementary genetic evidence comes from CTLA-4-transgenic *ApoE*−/− mice, in which CTLA-4 overexpression diminished atherosclerotic lesion formation and reduced intraplaque macrophage and CD4+ T-cell accumulation, together with reduced expression of the costimulatory ligands CD80 and CD86 themselves on lesional DCs, suggesting a feedback loop in which effective coinhibition also limits costimulatory ligand density [70].

### 4.3. Antibody-Mediated CTLA-4 Blockade: Relevance to Cancer Immunotherapy

Conversely, antibody-mediated CTLA-4 blockade—modeling the anticancer drug ipilimumab—aggravates atherosclerotic plaque inflammation and progression in hyperlipidemic mice, increasing plaque T-cell content and inflammatory macrophage markers [71]. This parallels clinical and imaging evidence that immune checkpoint inhibitors (ICIs) targeting CTLA-4 or PD-1/PD-L1 are associated with accelerated atherosclerotic events, arterial PET inflammation, and, more rarely, myocarditis [28,71,72]. Because CD80 sits at the intersection of the CTLA-4 and, via cis-heterodimerization, PD-L1/PD-1 axes, this ICI-associated vascular toxicity is a large-scale human corroboration of the atheroprotective role of CD80/CD86-dependent coinhibition identified in mouse genetics [34,73].

### 4.4. Direct Pharmacological Targeting of CD80 Itself

Beyond CTLA-4-Ig, which blocks CD80 and CD86 indiscriminately, at least one study has tested selective pharmacological inhibition of CD80 (B7-1) alone. RhuDex, a small-molecule B7-1 inhibitor, significantly reduced LPS-induced cytokine/chemokine release (TNF-α, IL-6, CCL2) from ex vivo human carotid endarterectomy plaque cultures and reduced T-cell activation in APC–T-cell cocultures, with effects mapped to NF-κB, AP-1 and ERK1/2 signaling in monocytes/macrophages—direct human-tissue evidence that CD80 specifically, independent of CD86, is a tractable anti-inflammatory target in plaque [74].

### 4.5. Dendritic-Cell-Restricted and Antigen-Specific Approaches

Because global CD80/CD86 blockade carries a risk of systemic immunosuppression, several groups have explored more selective, antigen-directed strategies that indirectly modulate CD80/CD86 density on APCs, building on the recognition that immature, CD80-low/CD86-low DCs are intrinsically tolerogenic while mature CD80+/CD86+ DCs favor effector T-cell priming [75]. Tolerogenic DC-based immunotherapy, in which autologous DCs are loaded with atherosclerosis-relevant antigens (oxLDL, ApoB100 peptides, HSP60) under tolerizing conditions (IL-10, vitamin D3 analogs, or pharmacological NF-κB inhibition) to yield a CD80low/CD86low/PD-L1high phenotype, represents a precision-medicine approach that could in principle achieve antigen-specific tolerance while sparing systemic immune competence [76,77]. A related bispecific strategy, fusing an anti-CD86 antibody to IL-10, selectively delivers tolerogenic signaling to CD86-expressing APCs to induce antigen-specific regulatory T cells, illustrating an alternative, receptor-targeted route to the same CD80/CD86low tolerogenic endpoint that could in principle be adapted to atherosclerosis-relevant antigens [78]. Statins independently reduce oxLDL-induced DC maturation (lower CD80, CD83 and CD86) and the subsequent T-cell activation this maturation supports, an effect that is at least partly mediated through microRNA let-7c and reversed by exogenous mevalonate, linking a widely used clinical therapy mechanistically to the CD80/CD86 axis [79,80]. Independent work confirms that simvastatin/atorvastatin reduce CD40, CD83, CD86 and HLA-DR on monocyte-derived DCs [81], that atorvastatin suppresses angiotensin-II-induced CD80/CD83/CD86 upregulation via PI3K/Akt/Nrf2 signaling [82], and that statins impair CD1d-restricted lipid antigen presentation via prenylation-dependent suppression of CD40, CD80 and CD86 on macrophages, lymphocytes and endothelial cells—together indicating convergent, mevalonate-linked routes by which this drug class dampens CD80-dependent costimulation [83].

## 5. CD80 in Human Atherosclerotic Disease

### 5.1. Plaque Transcriptomic and Single-Cell Evidence

Bulk and single-cell transcriptomic profiling of human plaques has repeatedly identified CD80 among the costimulatory and immune-checkpoint transcripts differentially expressed between high- and low-risk, or symptomatic and asymptomatic, lesions, together with CD28, ICOSLG and multiple coinhibitory receptors, underscoring that costimulatory pathway activity, not merely leukocyte density, distinguishes vulnerable from stable plaque phenotypes [84]. A dedicated single-cell RNA-sequencing atlas of the immune-checkpoint landscape in human atherosclerotic plaques found that CD86 was broadly expressed across monocyte, macrophage and DC clusters and interacts with both CD28 and CTLA-4, whereas CD80 was restricted to fewer myeloid clusters, suggesting a comparatively more discrete, possibly subset- or maturation-stage-specific, role for CD80 within the human plaque immune-checkpoint network relative to CD86 or PD-L1 [25]. This finding is important for the field because it tempers the assumption—largely derived from mouse studies in which CD80 and CD86 are often manipulated together—that CD80 is uniformly and abundantly expressed across the human plaque myeloid compartment; instead, CD80 expression appears concentrated in specific, more mature or activated subsets such as CCR7+FSCN1+DCs [7,25].

### 5.2. Correlation with Plaque Vulnerability

Independent of transcriptomic breadth, the magnitude of CD80/CD83/CD86 expression correlates with clinically relevant plaque phenotypes. In human carotid endarterectomy specimens, CD11c, CD80, CD83 and CD86 mRNA levels are significantly elevated in vulnerable, symptomatic plaques compared with stable, asymptomatic plaques, with the difference being most marked for mature DC markers [46]. CD80/CD86-targeted molecular imaging using radiolabeled belatacept (a CTLA-4-Ig-based fusion protein with high affinity for CD80/CD86) demonstrated binding in human carotid plaque sections that correlated with immune-cell infiltration and the presence of a large lipid/necrotic core—histological hallmarks of unstable plaque—supporting CD80/CD86 density as a candidate in vivo biomarker of plaque inflammatory activity [46].

### 5.3. Soluble CD80 and Systemic Biomarkers

A soluble form of CD80 (sCD80), generated by alternative splicing or proteolytic shedding, circulates in plasma and has been studied as a biomarker in several chronic inflammatory and autoimmune conditions, including systemic lupus erythematosus, myasthenia gravis and minimal-change glomerular disease, in which sCD80 correlates with disease activity or comorbidity burden [85,86,87]. In elderly cohorts, elevated plasma sCD80 (with soluble CTLA-4) is independently associated with higher comorbidity burden, consistent with sCD80 marking chronic, age-related immune activation (“inflammaging”) relevant to atherosclerotic risk [86]. Outside the cardiovascular field, tumoral CD80/CD86 co-expression correlates with improved survival in nasopharyngeal carcinoma, illustrating that CD80 density can index a more actively regulated tissue immune microenvironment, an analogy that may extend to plaque [88]. Although dedicated large-scale studies of circulating sCD80 as a cardiovascular biomarker are still comparatively sparse relative to more established markers such as soluble CD40 ligand, the biological rationale—reflecting systemic APC activation state and potentially serving as a decoy that modulates CD28/CTLA-4 signaling—parallels that already established for sCD40L in atherosclerotic disease and represents a plausible avenue for future biomarker development specific to the CD80 pathway [89,90].

## 6. CD80-Targeted Molecular Imaging of Atherosclerosis

Because CD80 (with CD86) is upregulated specifically on activated, lesion-infiltrating APCs rather than resting leukocytes, it is an attractive target for non-invasive imaging of plaque inflammatory activity, complementing established tracers such as 18F-FDG (macrophage glycolysis), 18F-sodium fluoride (microcalcification) and RGD-based integrin αvβ3 ligands [91,92,93,94]. In a shear-stress-induced carotid model in *ApoE*−/− mice [95], CD80 was upregulated in developing plaques, and two CD80-targeting PET radiotracers were evaluated alongside 18F-FDG and 18F-fluoro-D-mannose, establishing feasibility for CD80-specific PET imaging [94]. Indium-111-labeled belatacept accumulated specifically and displaceably in *ApoE*−/− plaques by SPECT and bound human carotid plaque sections correlating with immune infiltration and necrotic-core burden [46]. Copper-64-labeled abatacept visualized local APC activation after subcutaneous LPS challenge, with sequential CD80+/CD86+ cell increases followed by a later CTLA-4 rise, illustrating temporal dynamics relevant to longitudinal plaque monitoring [96]. Dedicated small-molecule PET ligands with high affinity for human CD80 have also been synthesized, an initial step toward non-antibody-based CD80 tracers with more favorable pharmacokinetics than large fusion-protein probes [97].

## 7. CD80 in the Context of Immune Checkpoint Inhibitor-Associated Cardiovascular Toxicity

The clinical use of ICIs in oncology has provided an unanticipated, large-scale test of the atherosclerosis immune-checkpoint hypothesis. CTLA-4 antibodies (e.g., ipilimumab) prevent CTLA-4 engagement of CD80/CD86, permitting unrestrained CD28-mediated costimulation, while PD-1/PD-L1 antibodies remove PD-1-mediated and, via disruption of the PD-L1:CD80 cis-heterodimer, CD80-mediated inhibitory signaling [41,98]. Cohort and imaging studies show that ICI therapy is associated with increased arterial PET/CT inflammation, accelerated coronary calcification, and increased major adverse cardiovascular events, alongside the rarer complication of ICI-associated myocarditis, in which α-myosin-specific CD8+ T cells expand and infiltrate myocardium following combined PD-1/CTLA-4 blockade in murine models [34,72,73]. Preclinical ICI cardiotoxicity models specifically implicate loss of CD80/CD86-CTLA-4-dependent coinhibition, mirroring the CTLA-4-transgenic and antibody-blockade atherosclerosis data of Section 4 [70]. A dedicated analysis of PD-1 and CTLA-4 in cardiovascular complications leading to heart failure similarly found that PD-1/PD-L pathway loss amplifies atherosclerosis in animal models and cataloged clinical myocardial fibrosis, autoimmune myocarditis and pericarditis from ipilimumab, nivolumab and pembrolizumab [99]. These findings carry two practical implications: cardiovascular surveillance should be incorporated into ICI management of at-risk patients, and the CD80-centered pathway offers a template for vascular-restricted (rather than systemic) modulators that could dampen atherogenic costimulation without compromising anti-tumor immunity, or protect against ICI-associated vascular toxicity without blunting oncologic efficacy [28,72].

## 8. Translational and Therapeutic Considerations

### 8.1. Repurposing Approved CD80/CD86-Targeted Biologics

Abatacept and its higher-affinity derivative belatacept are already approved for rheumatoid arthritis and renal transplantation, respectively, providing a substantial existing human safety record that could accelerate translational studies of CD80/CD86 blockade in atherosclerosis [46,67]. Observational pharmacoepidemiological studies in RA patients—a population with markedly elevated atherosclerotic risk independent of traditional risk factors—have found abatacept associated with lower cardiovascular event risk than TNF inhibitors (TNFi). A propensity-matched Medicare/MarketScan cohort reported a 20% reduction in composite cardiovascular risk with abatacept versus TNFi [100], a benefit most pronounced in patients with comorbid diabetes (hazard ratio 0.74) [101], and a national cohort switching from TNFi found abatacept associated with lower stroke, heart failure and composite major adverse cardiac event risk than rituximab [102]. A three-year multicenter observational study of carotid atherosclerosis in abatacept-treated RA patients found decelerated plaque progression regardless of age, with no new safety signals [103]. No randomized controlled trial has yet directly tested CD80/CD86 blockade as a therapy for atherosclerosis or its clinical sequelae, and the abatacept-versus-TNFi comparisons above derive entirely from observational RA cohorts; this distinction should be borne in mind when weighing the strength of the cardiovascular signal. While these data derive from an autoimmune disease population and are subject to the inherent limitations of observational pharmacoepidemiology (confounding by indication, channeling bias), they represent the closest existing clinical analog to a “natural experiment” testing CD80/CD86 blockade for atherosclerosis prevention and are consistent with, and mechanistically continuous with, the preclinical genetic and pharmacological data reviewed in Section 4 [104,105]. The results of key studies connected with drugs targeting CD80 are summarized in Table 2.

### 8.2. Selective CD80 Antagonism Versus Dual CD80/CD86 Blockade

A recurring theme of this review is that CD80 and CD86, despite sharing receptors, are not interchangeable, with direct implications for drug design. Broad CTLA-4-Ig blockade of both ligands (abatacept, belatacept) reduces atherosclerosis-related outcomes in mice and RA cohorts, but by depriving Tregs of CD86-dependent CD28 costimulation risks attenuating atheroprotective regulatory responses [35,67]. Selective CD80 antagonists such as RhuDex, by contrast, spare CD86–CD28 Treg support while blocking the CD80-dominant, high-avidity interactions implicated in effector priming and NF-κB/AP-1/ERK-driven monocyte activation, and they already reduce inflammatory mediator release from human plaque explants [74]. This distinction favors selective CD80 (rather than pan-B7) antagonism for chronic, long-term atherosclerosis therapy.

### 8.3. Antigen-Specific and Cell-Based Strategies

An alternative to systemic receptor blockade is to modulate CD80/CD86 expression selectively on antigen-specific APCs. Tolerogenic DC-based immunotherapy, in which autologous DCs are loaded with atherosclerosis-relevant antigens (oxLDL, ApoB100 peptides, HSP60) under tolerizing conditions (IL-10, vitamin D3 analogs, or pharmacological NF-κB inhibition) to yield a CD80low/CD86low/PD-L1high phenotype, represents a precision-medicine approach that could in principle achieve antigen-specific tolerance while sparing systemic immune competence [76,77]. Statins, already a cornerstone of atherosclerosis pharmacotherapy, contribute an ancillary, mevalonate-pathway-dependent suppression of DC CD80/CD83/CD86 maturation that may partly underlie their well-documented pleiotropic anti-inflammatory effects, independent of LDL-cholesterol lowering [79,80,82].

### 8.4. Diagnostic Applications

Beyond therapeutics, CD80-targeted imaging agents (radiolabeled belatacept, small-molecule CD80 PET ligands) could in principle be developed into clinical tools for non-invasive plaque phenotyping, potentially identifying rupture-prone, immunologically “hot” lesions that carry disproportionate risk of acute coronary or cerebrovascular events, complementing anatomical imaging (CT angiography, intravascular ultrasound, and emerging radionuclide immune-checkpoint tracers developed for oncology [106]) with a functional, immune-activity readout [46,94,97]. Such tools could additionally serve as pharmacodynamic biomarkers in early-phase trials of CD80/CD86-targeted atherosclerosis therapeutics, tracking target engagement and biological response ahead of hard cardiovascular endpoints.

## 9. Limitations and Open Questions

Several gaps constrain current understanding. First, most mechanistic and genetic data derive from murine models (*ApoE*−/−, *Ldlr*−/−, ApoE3*Leiden) and femoral-cuff remodeling models, which may not fully recapitulate the human plaque myeloid landscape, given the human-specific restriction of CD80 to discrete myeloid subsets on single-cell sequencing [25]. Second, most pharmacological interventions tested (CTLA-4-Ig, anti-CTLA-4 antibodies) act on CD80 and CD86 simultaneously, so CD80’s contribution independent of CD86 remains incompletely resolved outside the single RhuDex human-explant study [74]. Third, prospective human data directly linking circulating or plaque CD80 to hard cardiovascular endpoints are lacking; existing evidence is largely cross-sectional or derived indirectly from RA pharmacoepidemiology rather than dedicated atherosclerosis trials [84,100]. Fourth, the efficacy–safety balance for CD80-directed therapy—particularly the risk of impairing anti-tumor and anti-infective immunity, a documented consequence of CTLA-4-Ig therapy in transplantation and rheumatology—requires further definition for a chronic, lifelong disease requiring years-to-decades treatment [44,57]. Finally, the CD80–PD-L1 cis-heterodimer axis raises the possibility that PD-L1-targeted cancer therapies could have unintended, CD80-mediated effects on plaque biology not yet systematically characterized in humans [23,72]. More broadly, complementary efforts to integrate immune–inflammatory mechanisms with precision-medicine approaches across the atherosclerosis field further underscore the translational relevance of costimulatory pathways such as CD80 [107].

## 10. Conclusions

CD80 occupies a structurally and functionally distinct position within the B7 costimulatory family, engaging CD28, CTLA-4 and, through cis-heterodimerization, PD-L1 to integrate activating and inhibitory signals at the interface between innate and adaptive immunity in the atherosclerotic plaque. Genetic deletion, antibody blockade and CTLA-4-Ig-based pharmacological studies in mice, corroborated by human plaque transcriptomic, imaging and pharmacoepidemiological data, converge on a model in which CD80/CD86-dependent costimulation of effector T cells promotes atherogenesis and plaque instability, while CD80/CD86 engagement of CTLA-4 restrains this process—a balance that is disrupted, with measurable clinical consequences, by cancer immunotherapy targeting this same pathway. Selective small-molecule CD80 antagonism, CD80-targeted molecular imaging, and repurposing of existing CTLA-4-Ig biologics collectively represent a promising, though still early-stage, translational pipeline. Continued work to resolve the human-specific cell-type distribution of CD80, to dissect its contribution independent of CD86, and to establish prospective links between CD80 biomarkers and cardiovascular outcomes will be necessary to move this pathway from mechanistic insight toward clinical application in the prevention and treatment of atherosclerotic cardiovascular disease.

## Figures and Tables

**Table 2:** Selected preclinical and clinical studies targeting the CD80/CD86 costimulatory axis in atherosclerosis.

Intervention	Model/Population	Key Finding	Refs
CD80−/−CD86−/− genetic deletion	*Ldlr*−/− mice	Altered plaque HSP60-specific T-cell responses; regulates atherogenesis	[26]
CD4−/− and CD80−/−CD86−/−	Femoral artery cuff model	Significantly smaller intimal lesions vs. wild type	[67]
Abatacept (CTLA-4-Ig)	Femoral cuff model/ApoE3*Leiden mice	↓ 58.5% intimal thickening; ↓ 78.1% accelerated atherosclerosis	[67]
CTLA4-Ig	Homocysteine-accelerated *ApoE*−/− mice	Ameliorated atherosclerosis via reduced T-cell overactivation	[69]
CTLA-4 transgenic overexpression	*ApoE*−/− mice	↓ lesion formation; ↓ macrophage/CD4+ T-cell accumulation	[70]
Anti-CTLA-4 antibody blockade	Hyperlipidemic mice	Aggravated plaque inflammation/progression (models ipilimumab)	[71]
RhuDex (selective CD80 antagonist)	Human carotid endarterectomy plaque explants	↓ TNF-α, IL-6, CCL2 release; ↓ T-cell activation	[74]
Tolerogenic ApoB100-pulsed DCs (CD80low/CD86low)	Mouse atherosclerosis model	Induced Tregs; reduced lesion T-cell activation	[76]
Statins (atorvastatin/simvastatin)	Human monocyte-derived DCs/mouse models	↓ CD80/CD83/CD86 DC maturation markers	[79,81,82]
Abatacept (clinical)	RA patients, Medicare/MarketScan cohort	20% reduction in composite cardiovascular risk vs. TNFi	[100]
Abatacept (clinical)	RA patients with comorbid diabetes	Hazard ratio 0.74 for cardiovascular events vs. TNFi	[101]
Abatacept (clinical)	3-year observational carotid-atherosclerosis cohort	Decelerated carotid plaque progression, all ages	[103]
111In-belatacept SPECT/64Cu-abatacept PET	*ApoE*−/− mice; human plaque sections	Specific plaque binding correlating with immune infiltrate/necrotic core	[46,96]

RA, rheumatoid arthritis; TNFi, tumor necrosis factor inhibitor. Arrows denote directional change reported by the cited study.

## Data Availability

No new data were created or analyzed in this study. Data sharing is not applicable to this article.
